# Free-living clinging flatworms (Rhabditophora, Polycladida) associated with *Sargassum* from the Caribbean Coast of Colombia

**DOI:** 10.3897/BDJ.13.e150699

**Published:** 2025-05-07

**Authors:** Jorge I Merchán Mayorga, Sigmer Y Quiroga, Isabella Posada-Restrepo, Katherin A García-Ramos

**Affiliations:** 1 Universidad del Magdalena, Santa Marta, Colombia Universidad del Magdalena Santa Marta Colombia; 2 Universidad del Sinú, Cartagena, Colombia Universidad del Sinú Cartagena Colombia

**Keywords:** Corales de Profundidad National Natural Park, Corales del Rosario y San Bernardo National Natural Park, Platyhelminthes, Acotylea

## Abstract

**Background:**

Polyclads are a diverse group of marine free-living flatworms, with some species adapted to life in floating *Sargassum* mats. Recent studies suggest that, rather than being inherently pelagic, these flatworms should be classified as "clinging fauna", as they rely on floating substrates for habitat.

**New information:**

This study documents, for the first time, the occurrence of *Gnesiocerossargassicola* and *Chatziplanagrubei* in *Sargassum* along the Caribbean coast of Colombia. High-definition photographs of whole mounts and histological sections are provided for both species, along with detailed observations of their reproductive structures and 28S rDNA barcodes. These findings underscore the importance of exploring the fauna associated with *Sargassum*, contributing to a better understanding of polyclad distribution and raising the number of recorded species for Colombia to 26.

## Introduction

Floating *Sargassum* plays a fundamental ecological role in marine ecosystems, providing a dynamic habitat that offers shelter, feeding and spawning grounds for a wide diversity of organisms (Coston-Clements et al. 1991, Trott et al. 2011, Helm 2021). The Sargasso Sea, located in the North Atlantic Ocean, is defined by four major ocean currents: the Gulf Stream to the west, the North Atlantic Current to the north, the Canary Current to the east and the North Equatorial Current to the south (Ardron et al. 2011, Johns et al. 2020). Within this unique ecosystem, *Sargassumnatans* (Linnaeus) Gaillon, 1828 and *Sargassumfluitans* (Børgesen) Børgesen, 1914, are the dominant holopelagic species, completing their entire life cycle while floating freely in the ocean, reproducing asexually through vegetative fragmentation, which allows them to form extensive and persistent mats that can span vast areas (Hunn et al. 2022). From the Sargasso Sea, these mats travel into the Caribbean through the passage between Cuba and Haiti, carried by currents and winds that facilitate their widespread dispersal. In the Colombian Caribbean, the *Sargassum* mats are further transported by the Panama–Colombia gyre and its countercurrents, eventually reaching ecologically significant areas such as the Parque Nacional Natural Corales de Profundidad (PNNCPR) and the Parque Nacional Natural Corales del Rosario y San Bernardo (PNNCRSB) (Bula Meyer et al. 1993, Dueñas Lagos et al. 2017).

Seasonal fluctuations in *Sargassum* biomass are notable, with peaks typically occurring between July and December. During this period, the macroalgae often accumulate along the coastline, either by stranding on beaches or forming dense aggregations in nearshore waters. While these events have ecological significance, providing habitats and transporting numerous marine species, they also have far-reaching economic and environmental consequences, particularly for coastal communities. Issues such as beach fouling, water quality degradation and disruptions to tourism and fishing industries have been documented, emphasising the need for a balanced understanding of *Sargassum*'s ecological roles and effective management (Trott et al. 2011, Chávez et al. 2020, Cabrera 2021, Vázquez-Delfín et al. 2024).

As a floating habitat, *Sargassum* supports an intricate trophic network, offering substrates for sessile organisms such as hydroids, bryozoans and anemones, while simultaneously harbouring diverse mobile invertebrates, including polychaetes, amphipods and flatworms (Helm 2021, Alleyne 2022). Amongst these, free-living flatworms (Polycladida) are notable for their ecological roles as predators and scavengers, contributing to nutrient cycling and serving as prey for higher trophic levels (Martin et al. 2021).

The ecological plasticity of Polycladida allows them to exploit a variety of microhabitats, including pelagic environments such as *Sargassum* mats, being the only habitat known for some of these species (Hyman 1939b, Faubel 1983, Faubel 1984, Prudhoe 1985). Despite this, studies on pelagic flatworms associated with floating *Sargassum* remain limited, with most focusing on their occurrence in oceanic ecosystems such as the Gulf of Mexico and the Sargasso Sea (Graff 1893, Hyman 1939b, Faubel 1984), leaving their distribution, diversity and ecological roles in the Caribbean coast largely unexplored.

Given the increasing prevalence of *Sargassum* mats due to anthropogenic nutrient enrichment and climate change (Wang et al. 2019, Cabrera 2021), understanding the associated faunal communities is critical. In particular, the role of free-living flatworms in these habitats, as both predators and prey, is essential for comprehending the ecological dynamics of *Sargassum*-based ecosystems. This study presents the first records of clinging Polycladida associated with floating *Sargassum* along the Caribbean coast of Colombia. In addition to species identification, it provides comprehensive data, including high-resolution images, molecular sequences and ecological observations. These contributions aim to improve our understanding of the biodiversity, distribution and ecological significance of these flatworms.

## Materials and methods

### Sampled area

Parque Nacional Natural Corales de Profundidad (PNNCPR) is located between coordinates 9°43'16.591"–10°7'30.277" N and 76°0'16.254"–76°17'41.091" W, approximately 32 km from the nearest continental point, the Barú Peninsula. This protected area spans 142,195.15 hectares, with depths ranging from 34 to 1,234 metres. Together with Parque Nacional Natural Corales del Rosario y San Bernardo (PNNCRSB), these protected areas serve as key conservation sites in the central Colombian Caribbean. Both parks are situated off the coasts of the Bolívar, Córdoba and Sucre Departments (Morales Giraldo et al. 2017).

### Sampling and sample processing

**Figure 1.**
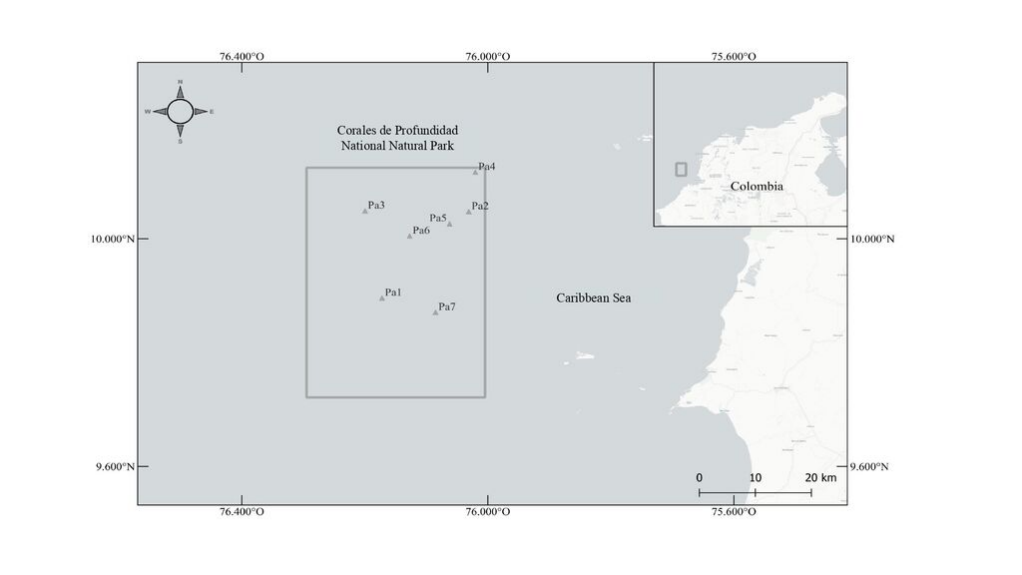
Study area and sampling points. Corales de Profundidad National Natural Park, central Colombian Caribbean. Sampling points are marked as Pa1, Pa2, Pa3, Pa4, Pa5, Pa6 and Pa7.

*Sargassum* patches within the study area were identified using satellite imagery from CoastWatch OceanView (NOAA) and SIMAR-SATsum (CONABIO). Sampling was conducted in 2022 and 2023. In 2022, one patch was located in May (Pa1) and two patches were identified in July (Pa2 and Pa3). In 2023, four patches were found in July (Pa4, Pa5, Pa6 and Pa7) (Fig. 1). Three samples were taken from each patch using a handheld net with a 1 mm mesh size (Muñoz Bautista et al. 2015). Each sample, approximately 2 kg of wet-weight *Sargassum*, was placed into buckets containing a magnesium sulphate and seawater solution (500 g:15 l) to narcotise polyclads (Williams and Van Syoc 2007). Sampling was conducted under the research permit "Resolución 043 de 2022" from CARDIQUE and with authorisation from Parques Nacionales Naturales de Colombia (PNNC). The samples were processed at the Aquaculture Laboratory of Universidad del Sinú, Cartagena. Manual agitation of the macroalgae was performed to dislodge the associated polyclad fauna. The resulting contents were filtered through a 150 μm sieve to isolate the macrofauna retained in the supernatant. The filtered material was placed in trays and organisms were manually separated using soft brushes. Subsequently, the specimens were examined under a Motic K-500L stereoscope. The organisms were quantified and grouped by morphotypes. Representative specimens of each morphotype were preserved in absolute ethanol for molecular analysis, while others were fixed and stored in 70% ethanol for whole mount preparations and histological processing. The remaining specimens were preserved in 70% ethanol.

### Taxonomic identifications

For some mature specimens of each species, the portion of the body containing the reproductive structures was dissected, dehydrated, embedded in paraffin and sagittally sectioned at a thickness of 5–7 μm for histological analysis. Serial histological sections were stained with haematoxylin and eosin and mounted on glass slides using Neo-Mount (Merck). Whole mount specimens were rehydrated, stained with haematoxylin and subsequently prepared by dehydrating the specimens, clearing them in methyl salicylate and mounting them in Neo-Mount. Taxonomic identifications followed the classification system of Faubel (1983) and the relevant literature containing the original species descriptions (Mertens 1832, Graff 1893). Classification and nomenclature were standardised using the World Register of Marine Species (WoRMS) database (2023). High-definition photographs of the sectioned and mounted specimens were obtained with a Zeiss AxioCam 208 colour camera mounted on an AxioLab 5 microscope and a Zeiss AxioCam ERc5s camera on a SteREO Discovery.V8 stereoscope. All specimens were deposited in the *Centro de Colecciones Científicas* of the Universidad del Magdalena, Santa Marta, Colombia (Catalogue CBUMAG:PLA).

### DNA barcoding

Total genomic DNA was extracted from each sample using an E.Z.N.A. Tissue DNA (Omega Bio-Tek) extraction kit following the manufacturer’s protocol. The 28S rDNA gene was amplified from whole-genomic DNA using MyTaq^TM^ DNA polymerase (Bioline), using the primer pair LSU_fw1 and LSU_rev2 for the D1-D2 region (Sonnenberg et al. 2007). The final PCR reaction volume was 25 μl each containing 3 μl of DNA template, 0.5 μl DNA polymerase (5 U/μl), 0.5 μl dNTP (10 mM), 1 μl of each primer (10 pmol), 1.5 μl MgCl_2_ (50 mM), 2.5 μl 10x Buffer and 15 μl ddH_2_O. PCR amplification conditions were as follows: initial denaturation at 95°C for 5 min, followed by 35 cycles of 95°C for 30 s, 55°C for 30 s and 72°C for 30 s, with a final extension at 72°C for 5 min. PCR amplification was confirmed by TBE gel electrophoresis in 2% agarose gel under UV light. The amplicons were sent to Macrogen Korea for purification and sequencing. Forward and reverse trace files were inspected using 4Peaks (Griekspoor and Groothuis 2015) and final sequences were edited and assembled in AliView (Larsson 2014). The obtained sequences of the D1-D2 28S region were compared with already published polyclad sequences using the BLAST tool from NCBI (Camacho et al. 2009).

## Taxon treatments

### Chatziplana grubei

(Graff, 1892)

6090E40B-D367-5F9A-974B-E550640AA23F

PQ962884

https://marinespecies.org/aphia.php?p=taxdetails&id=483712

Chatziplana grubei (Graff, 1892) Faubel 1983
Planocera grubei Graff, 1892
Hoploplana grubei (Graff, 1892)

#### Materials

- **Type status:** Other material. **Occurrence:** catalogNumber: CBUMAG:PLA:00627; preparations: whole mount (Neo-Mount); disposition: in collection; occurrenceID: F77FC249-FE7C-56B4-A797-F58CC75FD326; **Taxon:** scientificName: Chatziplanagrubei; taxonRank: species; **Location:** country: Colombia; decimalLatitude: 10.13538174; decimalLongitude: -76.00058405; **Record Level:** institutionID: 891.780.111-8; collectionID: RNC:207; institutionCode: Universidad del Magdalena (UniMagdalena); collectionCode: CBUMAG
- **Type status:** Other material. **Occurrence:** catalogNumber: CBUMAG:PLA:00628; preparations: whole mount (Neo-Mount); disposition: in collection; occurrenceID: 711937D3-85CC-56CB-90D5-388EB65BA9E5; **Taxon:** scientificName: Chatziplanagrubei; taxonRank: species; **Location:** country: Colombia; decimalLatitude: 10.13538174; decimalLongitude: -76.00058405; **Record Level:** institutionID: 891.780.111-8; collectionID: RNC:207; institutionCode: Universidad del Magdalena (UniMagdalena); collectionCode: CBUMAG
- **Type status:** Other material. **Occurrence:** catalogNumber: CBUMAG:PLA:00629; preparations: whole mount (Neo-Mount); disposition: in collection; occurrenceID: AEAD9DE2-EA50-5E7A-A560-C6625EA1B4E5; **Taxon:** scientificName: Chatziplanagrubei; taxonRank: species; **Location:** country: Colombia; decimalLatitude: 9.872; decimalLongitude: -76.086; **Record Level:** institutionID: 891.780.111-8; collectionID: RNC:207; institutionCode: Universidad del Magdalena (UniMagdalena); collectionCode: CBUMAG
- **Type status:** Other material. **Occurrence:** catalogNumber: CBUMAG:PLA:00630; preparations: stylet mount (Neo-Mount); disposition: in collection; occurrenceID: 0CBAB71B-01E2-5CAC-93BD-F3F1F073FB55; **Taxon:** scientificName: Chatziplanagrubei; taxonRank: species; **Location:** country: Colombia; decimalLatitude: 9.872; decimalLongitude: -76.086; **Record Level:** institutionID: 891.780.111-8; collectionID: RNC:207; institutionCode: Universidad del Magdalena (UniMagdalena); collectionCode: CBUMAG
- **Type status:** Other material. **Occurrence:** catalogNumber: CBUMAG:PLA:00631-1; preparations: Hematoxilin and eosin sagittal section 1-6; disposition: in collection; occurrenceID: 993E24C7-9891-5F57-8EEC-2A9E460B010D; **Taxon:** scientificName: Chatziplanagrubei; taxonRank: species; **Location:** country: Colombia; decimalLatitude: 10.13538174; decimalLongitude: -76.00058405; **Record Level:** institutionID: 891.780.111-8; collectionID: RNC:207; institutionCode: Universidad del Magdalena (UniMagdalena); collectionCode: CBUMAG
- **Type status:** Other material. **Occurrence:** catalogNumber: CBUMAG:PLA:00631-2; preparations: Hematoxilin and eosin sagittal section 2-6; disposition: in collection; occurrenceID: 0E0ECA14-F1FF-531D-98AE-3B6C58715C71; **Taxon:** scientificName: Chatziplanagrubei; taxonRank: species; **Location:** country: Colombia; decimalLatitude: 10.13538174; decimalLongitude: -76.00058405; **Record Level:** institutionID: 891.780.111-8; collectionID: RNC:207; institutionCode: Universidad del Magdalena (UniMagdalena); collectionCode: CBUMAG
- **Type status:** Other material. **Occurrence:** catalogNumber: CBUMAG:PLA:00631-3; preparations: Hematoxilin and eosin sagittal section 3-6; disposition: in collection; occurrenceID: 4CE71D39-04E5-5768-A40A-D52BD9747FE4; **Taxon:** scientificName: Chatziplanagrubei; taxonRank: species; **Location:** country: Colombia; decimalLatitude: 10.13538174; decimalLongitude: -76.00058405; **Record Level:** institutionID: 891.780.111-8; collectionID: RNC:207; institutionCode: Universidad del Magdalena (UniMagdalena); collectionCode: CBUMAG
- **Type status:** Other material. **Occurrence:** catalogNumber: CBUMAG:PLA:00631-4; preparations: Hematoxilin and eosin sagittal section 4-6; disposition: in collection; occurrenceID: 20EA8EE8-B2BC-5D37-A6BC-6DD96AE5DB0D; **Taxon:** scientificName: Chatziplanagrubei; taxonRank: species; **Location:** country: Colombia; decimalLatitude: 10.13538174; decimalLongitude: -76.00058405; **Record Level:** institutionID: 891.780.111-8; collectionID: RNC:207; institutionCode: Universidad del Magdalena (UniMagdalena); collectionCode: CBUMAG
- **Type status:** Other material. **Occurrence:** catalogNumber: CBUMAG:PLA:00631-5; preparations: Hematoxilin and eosin sagittal section 5-6; disposition: in collection; occurrenceID: 456CE3A8-CAAC-548E-9D35-5A96F3D13590; **Taxon:** scientificName: Chatziplanagrubei; taxonRank: species; **Location:** country: Colombia; decimalLatitude: 10.13538174; decimalLongitude: -76.00058405; **Record Level:** institutionID: 891.780.111-8; collectionID: RNC:207; institutionCode: Universidad del Magdalena (UniMagdalena); collectionCode: CBUMAG
- **Type status:** Other material. **Occurrence:** catalogNumber: CBUMAG:PLA:00631-6; preparations: Hematoxilin and eosin sagittal section 6-6; disposition: in collection; occurrenceID: 011ACD18-6A9A-5C0A-82DA-A81D9FA13D90; **Taxon:** scientificName: Chatziplanagrubei; taxonRank: species; **Location:** country: Colombia; decimalLatitude: 10.13538174; decimalLongitude: -76.00058405; **Record Level:** institutionID: 891.780.111-8; collectionID: RNC:207; institutionCode: Universidad del Magdalena (UniMagdalena); collectionCode: CBUMAG
- **Type status:** Other material. **Occurrence:** catalogNumber: CBUMAG:PLA:00632-1; preparations: Hematoxilin and eosin sagittal section 1-10; disposition: in collection; occurrenceID: 164D6A42-5564-578D-8DA4-8A1E3BD2CC8C; **Taxon:** scientificName: Chatziplanagrubei; taxonRank: species; **Location:** country: Colombia; decimalLatitude: 10.13538174; decimalLongitude: -76.00058405; **Record Level:** institutionID: 891.780.111-8; collectionID: RNC:207; institutionCode: Universidad del Magdalena (UniMagdalena); collectionCode: CBUMAG
- **Type status:** Other material. **Occurrence:** catalogNumber: CBUMAG:PLA:00632-2; preparations: Hematoxilin and eosin sagittal section 2-10; disposition: in collection; occurrenceID: 1AEFD29A-8BFB-5A9B-BC3C-C7A3B302D133; **Taxon:** scientificName: Chatziplanagrubei; taxonRank: species; **Location:** country: Colombia; decimalLatitude: 10.13538174; decimalLongitude: -76.00058405; **Record Level:** institutionID: 891.780.111-8; collectionID: RNC:207; institutionCode: Universidad del Magdalena (UniMagdalena); collectionCode: CBUMAG
- **Type status:** Other material. **Occurrence:** catalogNumber: CBUMAG:PLA:00632-3; preparations: Hematoxilin and eosin sagittal section 3-10; disposition: in collection; occurrenceID: 33991B30-6B8D-5C37-9FB5-FC2B8AC932E8; **Taxon:** scientificName: Chatziplanagrubei; taxonRank: species; **Location:** country: Colombia; decimalLatitude: 10.13538174; decimalLongitude: -76.00058405; **Record Level:** institutionID: 891.780.111-8; collectionID: RNC:207; institutionCode: Universidad del Magdalena (UniMagdalena); collectionCode: CBUMAG
- **Type status:** Other material. **Occurrence:** catalogNumber: CBUMAG:PLA:00632-4; preparations: Hematoxilin and eosin sagittal section 4-10; disposition: in collection; occurrenceID: 7BA30D76-E488-52C8-AEF8-6961E6271CB6; **Taxon:** scientificName: Chatziplanagrubei; taxonRank: species; **Location:** country: Colombia; decimalLatitude: 10.13538174; decimalLongitude: -76.00058405; **Record Level:** institutionID: 891.780.111-8; collectionID: RNC:207; institutionCode: Universidad del Magdalena (UniMagdalena); collectionCode: CBUMAG
- **Type status:** Other material. **Occurrence:** catalogNumber: CBUMAG:PLA:00632-5; preparations: Hematoxilin and eosin sagittal section 5-10; disposition: in collection; occurrenceID: 961181AD-DE40-588B-86C8-ADC23C1D0D49; **Taxon:** scientificName: Chatziplanagrubei; taxonRank: species; **Location:** country: Colombia; decimalLatitude: 10.13538174; decimalLongitude: -76.00058405; **Record Level:** institutionID: 891.780.111-8; collectionID: RNC:207; institutionCode: Universidad del Magdalena (UniMagdalena); collectionCode: CBUMAG
- **Type status:** Other material. **Occurrence:** catalogNumber: CBUMAG:PLA:00632-6; preparations: Hematoxilin and eosin sagittal section 6-10; disposition: in collection; occurrenceID: B2C1F620-5C22-5F9F-880B-98BF6BB87C7E; **Taxon:** scientificName: Chatziplanagrubei; taxonRank: species; **Location:** country: Colombia; decimalLatitude: 10.13538174; decimalLongitude: -76.00058405; **Record Level:** institutionID: 891.780.111-8; collectionID: RNC:207; institutionCode: Universidad del Magdalena (UniMagdalena); collectionCode: CBUMAG
- **Type status:** Other material. **Occurrence:** catalogNumber: CBUMAG:PLA:00632-7; preparations: Hematoxilin and eosin sagittal section 7-10; disposition: in collection; occurrenceID: 2DD63444-9A3B-5FD4-B42F-8EB1E249C84E; **Taxon:** scientificName: Chatziplanagrubei; taxonRank: species; **Location:** country: Colombia; decimalLatitude: 10.13538174; decimalLongitude: -76.00058405; **Record Level:** institutionID: 891.780.111-8; collectionID: RNC:207; institutionCode: Universidad del Magdalena (UniMagdalena); collectionCode: CBUMAG
- **Type status:** Other material. **Occurrence:** catalogNumber: CBUMAG:PLA:00632-8; preparations: Hematoxilin and eosin sagittal section 8-10; disposition: in collection; occurrenceID: 99394F20-3BCA-56A9-8F1C-B8415521E421; **Taxon:** scientificName: Chatziplanagrubei; taxonRank: species; **Location:** country: Colombia; decimalLatitude: 10.13538174; decimalLongitude: -76.00058405; **Record Level:** institutionID: 891.780.111-8; collectionID: RNC:207; institutionCode: Universidad del Magdalena (UniMagdalena); collectionCode: CBUMAG
- **Type status:** Other material. **Occurrence:** catalogNumber: CBUMAG:PLA:00632-9; preparations: Hematoxilin and eosin sagittal section 9-10; disposition: in collection; occurrenceID: 3CA52898-C10C-5DAF-847A-D57DFD973A18; **Taxon:** scientificName: Chatziplanagrubei; taxonRank: species; **Location:** country: Colombia; decimalLatitude: 10.13538174; decimalLongitude: -76.00058405; **Record Level:** institutionID: 891.780.111-8; collectionID: RNC:207; institutionCode: Universidad del Magdalena (UniMagdalena); collectionCode: CBUMAG
- **Type status:** Other material. **Occurrence:** catalogNumber: CBUMAG:PLA:00632-10; preparations: Hematoxilin and eosin sagittal section 10-10; disposition: in collection; occurrenceID: 2F628414-E89B-50E3-BCFB-1E8DD7CF54C0; **Taxon:** scientificName: Chatziplanagrubei; taxonRank: species; **Location:** country: Colombia; decimalLatitude: 10.13538174; decimalLongitude: -76.00058405; **Record Level:** institutionID: 891.780.111-8; collectionID: RNC:207; institutionCode: Universidad del Magdalena (UniMagdalena); collectionCode: CBUMAG
- **Type status:** Other material. **Occurrence:** catalogNumber: CBUMAG:PLA:00633-1; preparations: Hematoxilin and eosin sagittal section 1-6; disposition: in collection; occurrenceID: 79715431-F855-501A-B36A-2C942B70482D; **Taxon:** scientificName: Chatziplanagrubei; taxonRank: species; **Location:** country: Colombia; decimalLatitude: 9.872; decimalLongitude: -76.086; **Record Level:** institutionID: 891.780.111-8; collectionID: RNC:207; institutionCode: Universidad del Magdalena (UniMagdalena); collectionCode: CBUMAG
- **Type status:** Other material. **Occurrence:** catalogNumber: CBUMAG:PLA:00633-2; preparations: Hematoxilin and eosin sagittal section 2-6; disposition: in collection; occurrenceID: 969EE02A-9E5A-5E9F-8D83-D15963A4EE70; **Taxon:** scientificName: Chatziplanagrubei; taxonRank: species; **Location:** country: Colombia; decimalLatitude: 9.872; decimalLongitude: -76.086; **Record Level:** institutionID: 891.780.111-8; collectionID: RNC:207; institutionCode: Universidad del Magdalena (UniMagdalena); collectionCode: CBUMAG
- **Type status:** Other material. **Occurrence:** catalogNumber: CBUMAG:PLA:00633-3; preparations: Hematoxilin and eosin sagittal section 3-6; disposition: in collection; occurrenceID: C224F52E-03F7-54B2-BC42-45AC1190F85A; **Taxon:** scientificName: Chatziplanagrubei; taxonRank: species; **Location:** country: Colombia; decimalLatitude: 9.872; decimalLongitude: -76.086; **Record Level:** institutionID: 891.780.111-8; collectionID: RNC:207; institutionCode: Universidad del Magdalena (UniMagdalena); collectionCode: CBUMAG
- **Type status:** Other material. **Occurrence:** catalogNumber: CBUMAG:PLA:00633-4; preparations: Hematoxilin and eosin sagittal section 4-6; disposition: in collection; occurrenceID: 3C193E9B-A4CC-5C75-9607-55F5B195F15D; **Taxon:** scientificName: Chatziplanagrubei; taxonRank: species; **Location:** country: Colombia; decimalLatitude: 9.872; decimalLongitude: -76.086; **Record Level:** institutionID: 891.780.111-8; collectionID: RNC:207; institutionCode: Universidad del Magdalena (UniMagdalena); collectionCode: CBUMAG
- **Type status:** Other material. **Occurrence:** catalogNumber: CBUMAG:PLA:00633-5; preparations: Hematoxilin and eosin sagittal section 5-6; disposition: in collection; occurrenceID: F9ECD3BE-7BE6-5520-B690-C1E206E83272; **Taxon:** scientificName: Chatziplanagrubei; taxonRank: species; **Location:** country: Colombia; decimalLatitude: 9.872; decimalLongitude: -76.086; **Record Level:** institutionID: 891.780.111-8; collectionID: RNC:207; institutionCode: Universidad del Magdalena (UniMagdalena); collectionCode: CBUMAG
- **Type status:** Other material. **Occurrence:** catalogNumber: CBUMAG:PLA:00633-6; preparations: Hematoxilin and eosin sagittal section 6-6; disposition: in collection; occurrenceID: C209359F-E7A1-546C-BD03-F75F1C6F01FA; **Taxon:** scientificName: Chatziplanagrubei; taxonRank: species; **Location:** country: Colombia; decimalLatitude: 9.872; decimalLongitude: -76.086; **Record Level:** institutionID: 891.780.111-8; collectionID: RNC:207; institutionCode: Universidad del Magdalena (UniMagdalena); collectionCode: CBUMAG

#### Distribution

*Chatziplanagrubei* was first described from specimens collected in the Atlantic and Indian Oceans (Graff 1893) and was later recorded in the southern currents of Newfoundland, Canada (Plehn 1896) and in Saint Thomas, Virgin Islands (Bock 1913). It has been commonly found on *Sargassum* mats in the Sargasso Sea and the Gulf of Mexico (Hyman 1939b, Faubel 1984) and, more recently, in the Mexican Caribbean (van Tussenbroek et al. 2024b). Since its original description, no further records have been reported from the Indian Ocean. Here, we report for the first time the presence of *Chatziplanagrubei* in the central Colombian Caribbean associated with pelagic *Sargassum*.

#### Taxon discussion

Our specimens align with the description and illustrations from Graff (1893). Since its original description, the species has undergone two taxonomic changes. First, it was transferred, along with several species of *Planocera* Blainville, 1828 to *Hoploplana* by Laidlaw (1902), who established the genus due to its clear differences from the other members of *Planocera* that have a cylindrical penis with chitinous spines contrary to the species he transferred to *Hoploplana* which have a styliform penis; he also stated the presence of a muscular bursa copulatrix in *Planocera* contrary to the simpler female system of Hoploplanids. Later, Faubel (1983) re-assigned *Hoploplanagrubei* to the family Stylochocestidae Bock, 1913 and established the genus *Chatziplana* making it a monotypic genus, due to the ventral arrangement of the prostatic vesicle and the entire male reproductive system enclosed in a muscle bulb that no other Hoploplanid shares. Hence, both taxonomic changes were based mainly on the male reproductive structures of this species.

**Figure 2.**
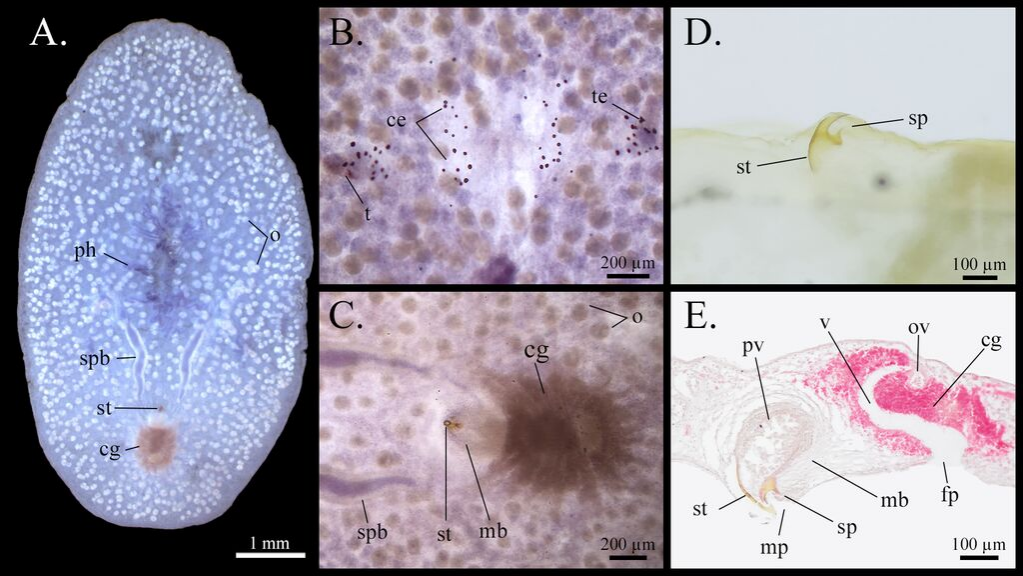
*Chatziplanagrubei* (Graff, 1892). **A** Whole mount; **B** Eyes detail; **C** Reproductive structures detail from whole mount; **D** Stylet preparation; **E** Sagittal section of reproductive structures. ce: cerebral eyes; cg: cement glands; fp: female pore; mb: muscular bulb; mp: male pore; fp: female pore; o: ovary; ov: oviduct; ph: pharynx; pv: prostatic vesicle; sp: spine; spb: spermiducal bulb; st: stylet; t: tentacle; te: tentacular eyes; v: vagina.

However, it is worth noting that, in Faubel's description, he mentions the presence of a flat seminal vesicle located dorsal to the prostatic vesicle. We did not observe any evidence of a seminal vesicle in our specimens. Instead, we consider the structure described by Faubel (1983) to be the cross-section where the spermiducal bulbs fuse and enter the prostatic vesicle (Fig. 2E). Additionally, we identified a previously unreported spine on the stylet of our specimens, located at the base of the stylet and curving in the same direction as the stylet point (Fig. 2D). This spine may have been difficult to observe in previous studies due to its small, thin and fragile structure, which could have easily been broken during specimens sectioning (Fig. 2E).

Regarding the female system, there is a contradiction in Faubel's monograph concerning the presence of Lang's vesicle. In the key to the genera of Stylochocestidae and the genus description of *Chatziplana*, the presence of a Lang's vesicle is mentioned. However, in his re-description of the copulatory apparatus of the species, Faubel states that the Lang's vesicle is absent –– an absence also reflected in his illustration. This inconsistency was carried over in the work of Bulnes et al. (2003), where two new genera were established for the family. The absence of this vesicle could result from an obervational oversight, as seen in *G.sargassicola* (see below). However, in Graff (1893)'s original description, he clearly states that the vagina leads only to a small bladder receiving the uteri from both sides and his illustrations do not depict a Lang's vesicle. We did not observe any evidence of a Lang's vesicle in our whole mounts or sagittal sections (Fig. 2).

We provide the first photographs of the reproductive structures and whole mounts, as well as the first genetic sequence for this species. In light of our observations, we consider that an update of Faubel's definition of the genus *Chatziplana* is warranted.

#### Emended Diagnosis Chatziplana Faubel, 1983

Stylochocestidae with tentacular and cerebral eyes. The body is of firm consistency. Tentacles present. Male and female pores separate. Male copulatory apparatus enclosed in a common muscular bulb arranged ventrally. Prostatic vesicle developed and distally enclosed by a pointed stylet with an accessory spine. At the dorsal base of the stylet, the ejaculatory duct opens into the prostatic vesicle. Developed spermiducal bulbs instead of a seminal vesicle. Female apparatus simple without Lang's vesicle.

Type of the genus: *Chatziplanagrubei* (Graff, 1892): With the characteristics of the genus.

### Gnesioceros sargassicola

(Mertens, 1833)

9F14D2ED-706D-5DD9-8979-7FFC423B0C5F

PQ962885

https://marinespecies.org/aphia.php?p=taxdetails&id=158296

Gnesioceros sargassicola (Mertens, 1833) Hyman 1939b
Gnesioceros mertens (Diesing, 1850)
Gnesioceros mertensi (Diesing, 1850)
Pelagoplana sargassicola (Mertens, 1833)
Planaria sargassicola Mertens, 1833
Planocera sargassicola (Mertens, 1833)
Stylochoplana sargassicola (Mertens, 1833)
Stylochus mertensi Diesing, 1850
Stylochus pelagicus Moseley, 1877
Stylochus pelagicus Moseley, 1877
Stylochus sargassicola (Mertens, 1833)

#### Materials

- **Type status:** Other material. **Occurrence:** catalogNumber: CBUMAG:PLA:00634; preparations: whole mount (Neo-Mount); disposition: in collection; occurrenceID: C03621CA-8BAA-519D-8584-1830479B4E58; **Taxon:** scientificName: Gnesiocerossargassicola; taxonRank: species; **Location:** country: Colombia; decimalLatitude: 10.13538174; decimalLongitude: -76.00058405; **Record Level:** institutionID: 891.780.111-8; collectionID: RNC:207; institutionCode: Universidad del Magdalena (UniMagdalena); collectionCode: CBUMAG; basisOfRecord: PreservedSpecimen
- **Type status:** Other material. **Occurrence:** catalogNumber: CBUMAG:PLA:00635; preparations: whole mount (Neo-Mount); disposition: in collection; occurrenceID: CCBB9BA2-3F3B-5B77-ADF5-BE8948FB394F; **Taxon:** scientificName: Gnesiocerossargassicola; taxonRank: species; **Location:** country: Colombia; decimalLatitude: 10.13538174; decimalLongitude: -76.00058405; **Record Level:** institutionID: 891.780.111-8; collectionID: RNC:207; institutionCode: Universidad del Magdalena (UniMagdalena); collectionCode: CBUMAG; basisOfRecord: PreservedSpecimen
- **Type status:** Other material. **Occurrence:** catalogNumber: CBUMAG:PLA:00636; preparations: whole mount (Neo-Mount); disposition: in collection; occurrenceID: 06BB7B3D-83A1-5DB2-B73C-925C1FDE1A59; **Taxon:** scientificName: Gnesiocerossargassicola; taxonRank: species; **Location:** country: Colombia; decimalLatitude: 10.13538174; decimalLongitude: -76.00058405; **Record Level:** institutionID: 891.780.111-8; collectionID: RNC:207; institutionCode: Universidad del Magdalena (UniMagdalena); collectionCode: CBUMAG; basisOfRecord: PreservedSpecimen
- **Type status:** Other material. **Occurrence:** catalogNumber: CBUMAG:PLA:00637-1; preparations: Hematoxilin and eosin sagittal section 1-3; disposition: in collection; occurrenceID: E1F34171-35D0-58B5-BE4A-DD14F111F5E8; **Taxon:** scientificName: Gnesiocerossargassicola; taxonRank: species; **Location:** country: Colombia; decimalLatitude: 10.13538174; decimalLongitude: -76.00058405; **Record Level:** institutionID: 891.780.111-8; collectionID: RNC:207; institutionCode: Universidad del Magdalena (UniMagdalena); collectionCode: CBUMAG; basisOfRecord: PreservedSpecimen
- **Type status:** Other material. **Occurrence:** catalogNumber: CBUMAG:PLA:00637-2; preparations: Hematoxilin and eosin sagittal section 2-3; disposition: in collection; occurrenceID: 387619E6-CF8E-5338-BBA9-7D2BEBDDA07A; **Taxon:** scientificName: Gnesiocerossargassicola; taxonRank: species; **Location:** country: Colombia; decimalLatitude: 10.13538174; decimalLongitude: -76.00058405; **Record Level:** institutionID: 891.780.111-8; collectionID: RNC:207; institutionCode: Universidad del Magdalena (UniMagdalena); collectionCode: CBUMAG; basisOfRecord: PreservedSpecimen
- **Type status:** Other material. **Occurrence:** catalogNumber: CBUMAG:PLA:00637-3; preparations: Hematoxilin and eosin sagittal section 3-3; disposition: in collection; occurrenceID: 86672D96-6D3C-5F84-89C8-26C9B8680B33; **Taxon:** scientificName: Gnesiocerossargassicola; taxonRank: species; **Location:** country: Colombia; decimalLatitude: 10.13538174; decimalLongitude: -76.00058405; **Record Level:** institutionID: 891.780.111-8; collectionID: RNC:207; institutionCode: Universidad del Magdalena (UniMagdalena); collectionCode: CBUMAG; basisOfRecord: PreservedSpecimen
- **Type status:** Other material. **Occurrence:** catalogNumber: CBUMAG:PLA:00638-1; preparations: Hematoxilin and eosin sagittal section 1-6; disposition: in collection; occurrenceID: 1F76A4DE-D9EF-5074-A58E-7ECD1E2871C1; **Taxon:** scientificName: Gnesiocerossargassicola; taxonRank: species; **Location:** country: Colombia; decimalLatitude: 10.13538174; decimalLongitude: -76.00058405; **Record Level:** institutionID: 891.780.111-8; collectionID: RNC:207; institutionCode: Universidad del Magdalena (UniMagdalena); collectionCode: CBUMAG; basisOfRecord: PreservedSpecimen
- **Type status:** Other material. **Occurrence:** catalogNumber: CBUMAG:PLA:00638-2; preparations: Hematoxilin and eosin sagittal section 2-6; disposition: in collection; occurrenceID: 778582C9-394E-590F-940D-EC9E7428FA9C; **Taxon:** scientificName: Gnesiocerossargassicola; taxonRank: species; **Location:** country: Colombia; decimalLatitude: 10.13538174; decimalLongitude: -76.00058405; **Record Level:** institutionID: 891.780.111-8; collectionID: RNC:207; institutionCode: Universidad del Magdalena (UniMagdalena); collectionCode: CBUMAG; basisOfRecord: PreservedSpecimen
- **Type status:** Other material. **Occurrence:** catalogNumber: CBUMAG:PLA:00638-3; preparations: Hematoxilin and eosin sagittal section 3-6; disposition: in collection; occurrenceID: B7AF631E-61A3-5762-B1B5-618B427367EA; **Taxon:** scientificName: Gnesiocerossargassicola; taxonRank: species; **Location:** country: Colombia; decimalLatitude: 10.13538174; decimalLongitude: -76.00058405; **Record Level:** institutionID: 891.780.111-8; collectionID: RNC:207; institutionCode: Universidad del Magdalena (UniMagdalena); collectionCode: CBUMAG; basisOfRecord: PreservedSpecimen
- **Type status:** Other material. **Occurrence:** catalogNumber: CBUMAG:PLA:00638-4; preparations: Hematoxilin and eosin sagittal section 4-6; disposition: in collection; occurrenceID: 5B2F493A-CA9C-5FD8-AF3E-ABAC4CA0F40B; **Taxon:** scientificName: Gnesiocerossargassicola; taxonRank: species; **Location:** country: Colombia; decimalLatitude: 10.13538174; decimalLongitude: -76.00058405; **Record Level:** institutionID: 891.780.111-8; collectionID: RNC:207; institutionCode: Universidad del Magdalena (UniMagdalena); collectionCode: CBUMAG; basisOfRecord: PreservedSpecimen
- **Type status:** Other material. **Occurrence:** catalogNumber: CBUMAG:PLA:00638-5; preparations: Hematoxilin and eosin sagittal section 5-6; disposition: in collection; occurrenceID: D51B89D0-E9BC-53A8-BAB3-8CFCE0F774DB; **Taxon:** scientificName: Gnesiocerossargassicola; taxonRank: species; **Location:** country: Colombia; decimalLatitude: 10.13538174; decimalLongitude: -76.00058405; **Record Level:** institutionID: 891.780.111-8; collectionID: RNC:207; institutionCode: Universidad del Magdalena (UniMagdalena); collectionCode: CBUMAG; basisOfRecord: PreservedSpecimen
- **Type status:** Other material. **Occurrence:** catalogNumber: CBUMAG:PLA:00638-6; preparations: Hematoxilin and eosin sagittal section 6-6; disposition: in collection; occurrenceID: D1435E05-C9BB-5742-8C1A-BA62B7C5075B; **Taxon:** scientificName: Gnesiocerossargassicola; taxonRank: species; **Location:** country: Colombia; decimalLatitude: 10.13538174; decimalLongitude: -76.00058405; **Record Level:** institutionID: 891.780.111-8; collectionID: RNC:207; institutionCode: Universidad del Magdalena (UniMagdalena); collectionCode: CBUMAG; basisOfRecord: PreservedSpecimen
- **Type status:** Other material. **Occurrence:** catalogNumber: CBUMAG:PLA:00639-1; preparations: Hematoxilin and eosin sagittal section 1-6; disposition: in collection; occurrenceID: 8B22DF88-F5C8-526C-99AF-EB80E8A7B078; **Taxon:** scientificName: Gnesiocerossargassicola; taxonRank: species; **Location:** country: Colombia; decimalLatitude: 10.13538174; decimalLongitude: -76.00058405; **Record Level:** institutionID: 891.780.111-8; collectionID: RNC:207; institutionCode: Universidad del Magdalena (UniMagdalena); collectionCode: CBUMAG; basisOfRecord: PreservedSpecimen
- **Type status:** Other material. **Occurrence:** catalogNumber: CBUMAG:PLA:00639-2; preparations: Hematoxilin and eosin sagittal section 2-6; disposition: in collection; occurrenceID: 0844E7DB-F128-5152-8F89-449A37A4507F; **Taxon:** scientificName: Gnesiocerossargassicola; taxonRank: species; **Location:** country: Colombia; decimalLatitude: 10.13538174; decimalLongitude: -76.00058405; **Record Level:** institutionID: 891.780.111-8; collectionID: RNC:207; institutionCode: Universidad del Magdalena (UniMagdalena); collectionCode: CBUMAG; basisOfRecord: PreservedSpecimen
- **Type status:** Other material. **Occurrence:** catalogNumber: CBUMAG:PLA:00639-3; preparations: Hematoxilin and eosin sagittal section 3-6; disposition: in collection; occurrenceID: D9C5D213-4A97-5DB5-9ABD-E2483B99DC8D; **Taxon:** scientificName: Gnesiocerossargassicola; taxonRank: species; **Location:** country: Colombia; decimalLatitude: 10.13538174; decimalLongitude: -76.00058405; **Record Level:** institutionID: 891.780.111-8; collectionID: RNC:207; institutionCode: Universidad del Magdalena (UniMagdalena); collectionCode: CBUMAG; basisOfRecord: PreservedSpecimen
- **Type status:** Other material. **Occurrence:** catalogNumber: CBUMAG:PLA:00639-4; preparations: Hematoxilin and eosin sagittal section 4-6; disposition: in collection; occurrenceID: AB452021-45C2-5185-8927-76B0EE97ED35; **Taxon:** scientificName: Gnesiocerossargassicola; taxonRank: species; **Location:** country: Colombia; decimalLatitude: 10.13538174; decimalLongitude: -76.00058405; **Record Level:** institutionID: 891.780.111-8; collectionID: RNC:207; institutionCode: Universidad del Magdalena (UniMagdalena); collectionCode: CBUMAG; basisOfRecord: PreservedSpecimen
- **Type status:** Other material. **Occurrence:** catalogNumber: CBUMAG:PLA:00639-5; preparations: Hematoxilin and eosin sagittal section 5-6; disposition: in collection; occurrenceID: B76C44F2-0A36-592D-A1CB-39130377CFFB; **Taxon:** scientificName: Gnesiocerossargassicola; taxonRank: species; **Location:** country: Colombia; decimalLatitude: 10.13538174; decimalLongitude: -76.00058405; **Record Level:** institutionID: 891.780.111-8; collectionID: RNC:207; institutionCode: Universidad del Magdalena (UniMagdalena); collectionCode: CBUMAG; basisOfRecord: PreservedSpecimen
- **Type status:** Other material. **Occurrence:** catalogNumber: CBUMAG:PLA:00639-6; preparations: Hematoxilin and eosin sagittal section 6-6; disposition: in collection; occurrenceID: 06F6E257-8876-5D73-891C-B2F8DE3B30C1; **Taxon:** scientificName: Gnesiocerossargassicola; taxonRank: species; **Location:** country: Colombia; decimalLatitude: 10.13538174; decimalLongitude: -76.00058405; **Record Level:** institutionID: 891.780.111-8; collectionID: RNC:207; institutionCode: Universidad del Magdalena (UniMagdalena); collectionCode: CBUMAG; basisOfRecord: PreservedSpecimen

#### Distribution

*Gnesiocerossargassicola* was first described by Mertens (1832) from specimens found on floating *Sargassum* in the Sargasso Sea. It has been recorded on *Sargassum* mats in the Gulf of Mexico, the Caribbean Sea and the Sargasso Sea (Plehn 1896, Hyman 1939b, Faubel 1983, Faubel 1984, van Tussenbroek et al. 2024a, van Tussenbroek et al. 2024b) and in the western and central regions of the North Atlantic, in Bermuda (Hyman 1954), Boa Vista in Cape Verde (Laidlaw 1906) and off the coast of West Africa (Moseley 1877).

Additionally, *G.sargassicola* has been found in littoral environments, including Devil's Foot Island, Quisset Harbor and Florida (Hyman 1939a, du Bois-Reymond Marcus and Marcus 1968), as well as in the Cayman Islands (Prudhoe 1944), Curaçao, Bonaire, Puerto Rico and Saint Barthelemy (du Bois-Reymond Marcus and Marcus 1968). More recently, it has been reported from Santa Marta, Colombia (Quiroga et al. 2004) and in the Canary Islands (Cuadrado et al. 2021). Here, we report for the first time the presence of *Gnesiocerossargassicola* in floating *Sargassum* mats off the central Caribbean coast of Colombia.

#### Taxon discussion

**Figure 3.**
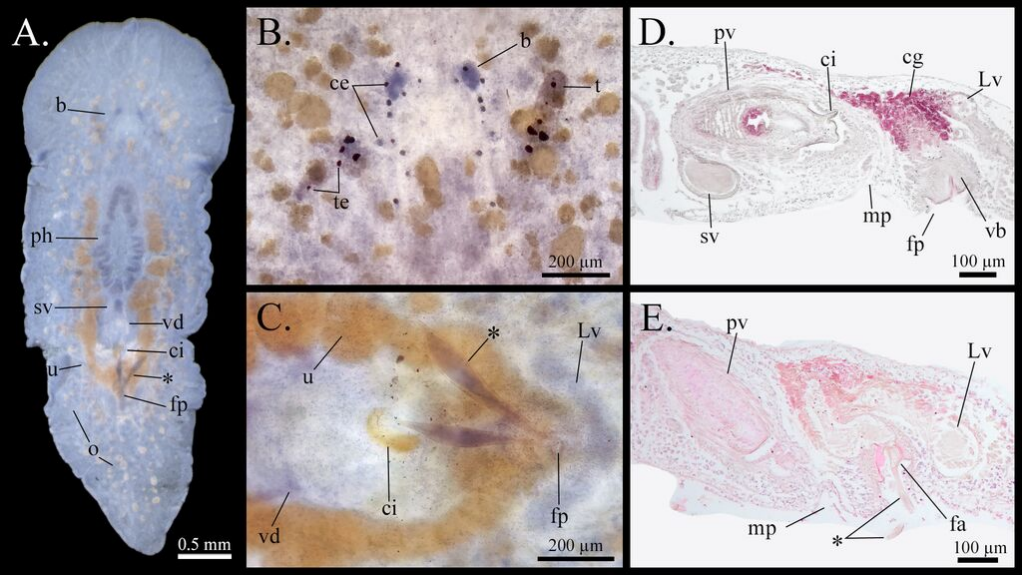
*Gnesiocerossargassicola* (Mertens, 1833). **A** Whole mount; **B** Eyes detail; **C** Reproductive structures detail from whole mount; **D, E** Sagittal sections of reproductive structures. b: brain; ce: cerebral eyes; cg: cement glands; ci: cirrus; fa: female atrium; fp: female pore; Lv: Lang's vesicle; mp: male pore; o: ovary; ph: pharynx; pv: prostatic vesicle; sv: seminal vesicle; t: tentacle; te; tentacular eyes; u: uteri; vb: vagina bulbosa; vd: vas deferens; *: "spermatophore".

Our specimens align well with the morphological descriptions provided by Hyman (1939b), du Bois-Reymond Marcus and Marcus (1968). As noted by du Bois-Reymond Marcus and Marcus (1968), our specimens are relatively small in comparison to the littoral specimens reported elsewhere. Hyman (1939b) also described G.sargassicolavar.lata Hyman, 1939 from seaweed roots in Bermuda, characterised by a broad anterior that quickly tapers to the posterior end; however, we did not find any specimen that shared this description. Remarkably, we also observed the "club-shaped" object attached to the female genital pore, as described by Hyman (1939b), in several specimens. We found between one and three of these objects in mature specimens. These structures are visible in both our whole mounts (Fig. 3A and C) and sagittal sections (Fig. 3D and E). They appear as elongated structures with a cuticular envelope resembling a honeycomb. While no internal structures or organs were observable in the histological sections, basophilic cells can be identified (Fig. 3C).

Hyman (1939b) suggested that the function of these structures may be related to reproduction, possibly as a secretion from the musculo-glandular ring in the female antrum. Our observations indicate that the cells inside these structures are spermatozoa, suggesting they might correspond to "spermatophores". This supports the findings of du Bois-Reymond Marcus and Marcus (1968), who reported a similar structure in one of their specimens from Curaçao. These "spermatophores" are likely deposited during copulation; however, to accurately define their nature, further research on the reproductive biology and mating behaviour of this species is necessary. Prudhoe (1985) referreed to these as "true" spermatophores because they are attached to the female pore, in contrast to similar-looking structures left behind in cases of hypodermic insemination in other polyclad species, which have also been called spermatophores. To our knowledge, no other true spermatophores have been reported in polyclads.

Faubel (1983) description of *G.sargassicola* provided a more comprehensive account of the reproductive structures of the species than previous works. However, a misinterpretation led him and others to believe that the Lang's vesicle was roundish. While the original description by Mertens (1832) is unclear on this point, Graff (1893) refers to it as an accessory vesicle in his work and incorporated it into his illustrations. However, due to the lack of clarity in Graff's figures, Hyman (1939b) subsequently demonstrated the transverse position of the Lang's vesicle. Our own observations of whole mounts (Fig. 3C) further corroborate the crescent-shape of Lang's vesicle. This feature supports the idea that its shape may serve as a valid taxonomic character within the family Gnesiocerotidae Marcus & Marcus, 1966, with the exception of *Comoplanaagilis* (Lang, 1884) (although see Oya and Kajihara (2020) for a more comprehensive discussion on the family's taxonomic status).

To better illustrate the relevant taxonomic characters of the species, we provide colour photographs of whole mounts and histological sections (Fig. 3). Additionally, we present the first genetic sequence of *G.sargassicola* from pelagic *Sargassum*.

## Analysis

### Samples description

**Figure 4.**
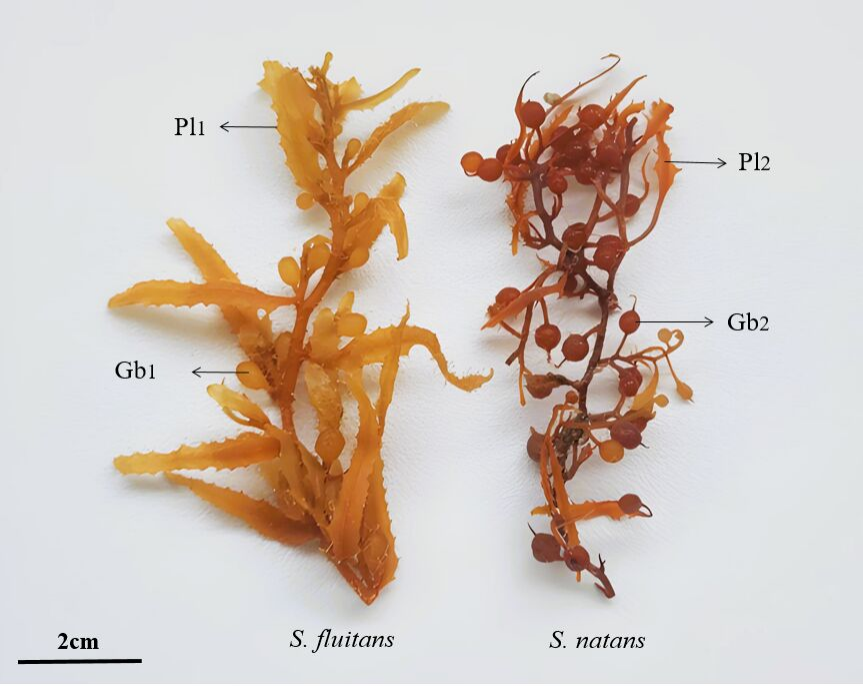
Species forming the floating mats of *Sargassum*. Left: *S.fluitans*, gas bladder without spine (Gb1), lanceolate phylloid with serrulate margin (Pl1), Right: *S.natans*, gas bladder with spine (Gb2), linear phylloid with serrulate margin (Pl2).

Two species of *Sargassum* were identified in the sampled floating beds: *Sargassumnatans* (Linnaeus) Gaillon, 1828 and *Sargassumfluitans* (Børgesen) Børgesen, 1914 (Fig. 4). Two species of polyclad flatworms were found clinging to these *Sargassum* species: *Chatziplanagrubei* (Graff, 1892) (Fig. 2) and *Gnesiocerossargassicola* (Mertens, 1833) (Fig. 3).

Polyclads were only present in the 2023 sampling, with a total of 174 individuals found. Of these, 134 belonged to *Chatziplanagrubei* and 40 to *Gnesiocerossargassicola*. The highest total abundance was observed in patch seven (Pa7), with 86 individuals of *C.grubei* and five of *G.sargassicola*. This was followed by patch four (Pa4), which contained 45 individuals of *C.grubei* and 30 of *G.sargassicola*. Patch five (Pa5) yielded three individuals of *C.grubei* and five of *G.sargassicola*. No polyclads were found in patch six (Pa6).

Other common clinging invertebrates in our samples included crustaceans such as bopyrids and caridean shrimps, litiopid snails and nereid polychaetes.

### DNA Barcoding

Partial sequences of the large ribosomal subunit (28S rDNA) gene were obtained and published under accession numbers PQ962884-PQ962885. Both sequences are approximately 1000 bp in length. The sequence of *Chatziplanagrubei* shows high similarity (> 94%) with members of the genus *Hoploplana* Laidlaw, 1902 and other members of the superfamily Stylochoidea Poche, 1926 (Table 1). Notably, no public sequence of the family Stylochocestidae Bock, 1913 is available; thus, the phylogenetic position inside the superfamily of the Stylochocestids and, thus, of *Chatziplanagrubei* has not been established through molecular analysis.

The BLAST results for the *Gnesiocerossargassicola* sequence showed 100% similarity to the only other published 28S sequence of *G.sargassicola*, originating from a littoral population in Colombia. Additionally, it is highly similar to other Gnesiocerotids and Stylochoplanids (Table 1).

## Discussion

According to Prudhoe (1985), approximately 20 species of polyclad flatworms have been reported floating in the ocean. However, only about 12 of these have been genuinely classified as 'pelagic' (Faubel 1984) (Table 2). Traditionally, the term pelagic has been used to describe species associated with floating algae. More recent studies, however, have re-defined this classification, referring to some organisms, including flatworms specifically associated with *Sargassum*, as 'clinging fauna' (Alleyne 2022). We consider this terminology more appropriate, as it reflects that flatworms themselves are not inherently pelagic, but rather inhabit floating substrates.

In this study, the only species of polyclad flatworms identified in association with *Sargassum* were *Gnesiocerossargassicola* and *Chatziplanagrubei*, both considered the dominant species in this habitat (Faubel 1984). Remarkably, although these species have been previously recorded in the Caribbean (Bock 1913, Hyman 1939b, Faubel 1983, van Tussenbroek et al. 2024b, van Tussenbroek et al. 2024a), this is the first time they have been documented on *Sargassum* along the coasts of Colombia. *Gnesiocerossargassicola* was previously reported on rocky shores in Santa Marta (Magdalena Department), but had not been associated with *Sargassum* (Quiroga et al. 2004). This study expands its known distribution to include the coasts of Bolívar and Córdoba Departments. Conversely, *Chatziplanagrubei* is recorded for the first time in Colombia, marking its southernmost occurrence in the Caribbean Sea. In addition, we provide new observations on the internal morphology of both species. Previous studies on polyclad biodiversity in Colombia had focused exclusively on the rocky shores of Magdalena, documenting 25 species in the Santa Marta Region (Quiroga et al. 2004). This research broadens the geographic scope of polyclad records to other regions along the Colombian Caribbean coast and introduces a new species to the national inventory, bringing the total to 26 formally documented species in Colombia.

According to Faubel (1984), *Gnesiocerossargassicola* is the dominant flatworm species in the Sargasso Sea, exhibiting remarkable thermal flexibility, as it thrives in both cold waters (as low as 2°C, for example, Woods Hole, USA) and warm waters (up to 25°C). In contrast, *Chatziplanagrubei*, also found in the Sargasso Sea, is strictly associated with *Sargassum* algae and limited to warm waters (≥ 20°C). Its distribution was previously thought to be more restricted, primarily within the Sargasso Sea gyre and the Gulf of Mexico. Our findings reveal that both species have extended their range to the Colombian Caribbean coast, likely facilitated by oceanic currents such as the Panama Gyre, the Caribbean Current and the Gulf Stream. These currents may have played a crucial role in dispersing these species more broadly across the Caribbean. However, their distribution could extend even further, driven by the increasing abundance of *Sargassum* in recent decades and the growing scientific attention to this phenomenon (Smetacek and Zingone 2013, Johns et al. 2020).

The proliferation of *Sargassum* has been linked to rising sea temperatures, nutrient inputs from the Amazon River and shifts in ocean circulation, contributing to the formation of the Atlantic *Sargassum* Belt, which stretches from the eastern coast of Africa to the Gulf of Mexico (Smetacek and Zingone 2013, Wang et al. 2019, Johns et al. 2020). The expansion of *Sargassum* habitats has not only reshaped the distribution of marine species, but has also introduced significant environmental and economic challenges (Smetacek and Zingone 2013, Chávez et al. 2020, Fidai et al. 2020, Vázquez-Delfín et al. 2024).

By documenting the presence of *Gnesiocerossargassicola* and *Chatziplanagrubei* in the central Colombian Caribbean, this study enhances our understanding of the biogeography of these flatworms. Furthermore, it highlights the influence of environmental factors and oceanic dynamics on their distribution. These findings underscore the need for more in-depth research to elucidate global distribution patterns and refine the taxonomy of these species.

## Supplementary Material

XML Treatment for Chatziplana grubei

XML Treatment for Gnesioceros sargassicola

## Figures and Tables

**Table 1.** BLAST Results.

***Chatziplanagrubei* - PQ962884**			***Gnesiocerossargassicola* - PQ962885**		
**Species**	**BLAST Identity** %	**Accession number**	**Species**	**BLAST Identity** %	**Accession number**
*Hoploplanadivae* Marcus, 1950	94.80%	KY263692.2	*Gnesiocerossargassicola* (Mertens, 1833)	100%	MH700309.1
*Hoploplanadivae* Marcus, 1950	94.78%	KY263693.2	*Ceratoplanafalconare* Rodriguez, Hutchings & Williamson, 2021	98.10%	MW377493.1
*Hoploplanacalifornica* Hyman, 1953	94.58%	KC869850.1	*Phaenoplanakopepe* Oya & Kajihara, 2019	97.50%	LC508133.1
*Hoploplanaornata* Yeri & Kaburaki, 1918	94.32%	LC508135.1	*Comoplanaagilis* (Lang, 1884)	97.39%	MN384685.1
*Heteroplanocerakatoi* Oya & Kajihara, 2021	93.13%	LC545568.1	*Styloplanocerafasciata* (Schmarda, 1859)	97.35%	MH700408.1
*Mirostylochusakkeshiensis* Kato, 1937	92.93%	LC508149.1	*Styloplanocerafasciata* (Schmarda, 1859)	97.34%	MH700409.1
Leptostylochuscf.gracilis Kato, 1934	92.73%	LC508138.1	*Stylochoplanaclara* Kato, 1937	96.97%	MW377494.1
*Neostylochusancorus* Rodriguez, Hutchings & Williamson, 2021	92.51%	MW377501.1	*Notocomplanaferruginea* (Schmarda, 1859)	94.94%	MT677877.1
*Paraplehniaseisuiae* Oya, Kimura & Kajihara, 2019	92.43%	LC467000.1	*Paraboliamegae* Rodriguez, Hutchings & Williamson, 2021	94.86%	MW377497.1
*Paraplehniapacifica* (Kato, 1939)	92.43%	LC508132.1	*Notoplana* sp. Laidlaw, 1903	94.62%	KY263651.2

**Table 2.** List of known species of clinging polyclads. O - Obligate: Found only associated with pelagic enviorments; F - Facultative: Found both in pelagic environments and other habitats; * - Incertae sedis. Note: *L.tremellaris* was mentioned by Graff (1893) from the *Sargassum* specimens he examined; however, he did not provide a description of its internal morphology. Modified from Faubel (1984).

Species of clinging polyclads	
*Acerotisanotulata* (Bosc, 1802)	O
*Chatziplanagrubei* (Graff, 1893)	O
*Coronadenamutabilis* (Verril, 1873)	F
*Gnesiocerossargassicola* (Mertens, 1833)	F
*Graffizoonlobatum* Heath, 1928*	O
*Leptoplanatremellaris* (Müller OF, 1773)	F
*Leptoplanellacalifornica* (Woodworth, 1894)	O
*Notoplehnianationalis* (Plehn, 1896)	F
*Phaenoplanachallengeri* (Graff, 1892)	F
*Planctoplanellaatlantica* Hyman, 1940	F
*Planocerapellucida* (Mertens, 1833) Örsted, 1844	F
*Prosthiostomumnationale* Plehn, 1896	F
*Pseudocerosvelutinus* (Blanchard, 1847) Lang, 1884	F

## References

[B12505059] Alleyne Kristie (2022). How is pelagic *Sargassum*-associated biodiversity assessed? Insights from the literature. Gulf and Caribbean Research.

[B12495401] Ardron Jeff, Halpin Pat, Roberts Jason, Cleary Jesse, Moffitt Russell, Donnelly Ben (2011). Where is the Sargasso Sea? A report submitted to the Sargasso Sea Alliance. http://sargasso.nonprofitsoapbox.com/storage/documents/No2_WhereistheSS_LO.pdf.

[B12494620] Bock Sixten (1913). Studien über polycladen. Zoologiska Bijdrag från Uppsala.

[B12493498] Bula Meyer Germán, Díaz Pulido Guillermo, Celis Rincón Argemiro (1993). Adiciones a las macroalgas de los arrecifes coralinos de las Islas del Rosario, con nuevos registros para el Caribe colombiano y el Atlántico. Boletín de Investigaciones Marinas y Costeras.

[B12700612] Bulnes Veronica N., Faubel Anno, Ponce de León Rodrigo (2003). New species of Stylochocestididae and Cryptocelididae (Plathelminthes, Polycladida: Acotylea) from the Atlantic coast of Uruguay. Mitteilungen aus dem Hamburgischen Zoologischen Museum und Institut, (Mitt. Hamb. Zool. Mus. Inst.).

[B12492990] Cabrera Rubén (2021). Registro de arribazón inusual de *Sargassum* (Phaeophyceae) para la costa Atlántica de Costa Rica. Hidrobiológica.

[B12505047] Camacho Christiam, Coulouris George, Avagyan Vahram, Ma Ning, Papadopoulos Jason, Bealer Kevin, Madden Thomas L. (2009). BLAST+: architecture and applications. BMC Bioinformatics.

[B12492787] Chávez Valeria, Uribe-Martínez Abigail, Cuevas Eduardo, Rodríguez-Martínez Rosa E., van Tussenbroek Brigitta I., Francisco Vanessa, Estévez Miriam, Celis Lourdes B., Monroy-Velázquez L. Verónica, Leal-Bautista Rosa, Álvarez-Filip Lorenzo, García-Sánchez Marta, Masia Luis, Silva Rodolfo (2020). Massive influx of pelagic *Sargassum* spp. on the coasts of the Mexican Caribbean 2014–2020: Challenges and opportunities. Water.

[B12492820] Coston-Clements Linda, Settle Lawrence R., Hoss Donald E., Cross Ford A. (1991). Utilization of the *Sargassum* habitat by marine invertebrates and vertebrates, a review.

[B12495056] Cuadrado Daniel, Rodríguez Jorge, Moro Leopoldo, Grande Cristina, Noreña Carolina (2021). Polycladida (Platyhelminthes, Rhabditophora) from Cape Verde and related regions of Macaronesia. European Journal of Taxonomy.

[B12495038] du Bois-Reymond Marcus Eveline, Marcus Ernst (1968). Polycladida from Curaçao and faunistically related regions. Studies on the Fauna of Curaçao and other Caribbean Islands.

[B12493507] Dueñas Lagos A., Bastidas Salamanca M., Ricaurte Villota C., Ricaurte Villota C., Bastidas Salamanca M. (2017). Regionalización oceanográfica: una visión dinámica del Caribe.

[B12493565] Faubel A. (1983). The Polycladida, Turbellaria. Proposal and establishment of a new system. Part I. The Acotylea. Mitteilungen aus dem Zoologischen Museum, Hamburg.

[B12492686] Faubel A. (1984). On the geographical occurrence of pelagic polyclad turbellarians. Cahiers de Biologie Marine.

[B12492704] Fidai Y. A., Dash J., Tompkins E. L., Tonon T. (2020). A systematic review of floating and beach landing records of *Sargassum* beyond the Sargasso Sea. Environmental Research Communications.

[B12492695] Graff L. von (1893). Pelagische polycladen. Z wiss Zool.

[B12493601] Griekspoor A., Groothuis T. (2015). 4Peaks. https://nucleobytes.com/4peaks/.

[B12495392] Helm Rebecca R. (2021). Natural history of neustonic animals in the Sargasso Sea: reproduction, predation, and behavior of *Glaucusatlanticus*, *Velellavelella*, and *Janthina* spp.. Marine Biodiversity.

[B12493520] Hunn Dayna, Blanar Christopher, Kerstetter David W. (2022). Evidence of spatial stability in core fauna community structure of holopelagic *Sargassum*. Caribbean Journal of Science.

[B12504990] Hyman Libbie H. (1939). Some polyclads of the New England coast, especially of the Woods Hole region. Biological Bulletin.

[B12495020] Hyman Libbie H. (1939). Acoel and polyclad Turbellaria from Bermuda and the *Sargassum*. Bulletin of The Bingham Oceanographic Collection.

[B12494928] Hyman Libbie Henrietta (1954). Free-living flatworms (Turbellaria) of the Gulf of Mexico. Fishery bulletin. United States Fish and Wildlife Service.

[B12492828] Johns Elizabeth M., Lumpkin Rick, Putman Nathan F., Smith Ryan H., Muller-Karger Frank E., T. Rueda-Roa Digna, Hu Chuanmin, Wang Mengqiu, Brooks Maureen T., Gramer Lewis J., Werner Francisco E. (2020). The establishment of a pelagic *Sargassum* population in the tropical Atlantic: Biological consequences of a basin-scale long distance dispersal event. Progress in Oceanography.

[B12496565] Laidlaw F. F., Gardiner J. S. (1902). University Press, Cambridge.

[B12494937] Laidlaw Frank Fortescue (1906). On the marine fauna of the Cape Verde islands from collections made in 1904 by Mr. C. Crossland: The polyclad Turbellaria. Proceedings of the Zoological Society of London.

[B12494552] Larsson Anders (2014). AliView: a fast and lightweight alignment viewer and editor for large datasets. Bioinformatics.

[B12493008] Martin Lindsay M., Taylor Madalyn, Huston Grayson, Goodwin Deborah S., Schell Jeffrey M., Siuda Amy N. S. (2021). Pelagic *Sargassum* morphotypes support different rafting motile epifauna communities. Marine Biology.

[B12493574] Mertens H. (1832). Untersuchungen über den innern bau verschiedener in der see lebender planarien.. Mémoires de l'Académie Impériale des Sciences de St. Pétersbourg. 6e série, Sciences Mathématiques, Physiques et Naturelles.

[B12493530] Morales Giraldo David Fernando, Rocha Gutiérrez Venus Lorena, Posada Posada Blanca Oliva (2017). Geomorfología de los fondos submarinos del Parque Nacional Natural Corales de Profundidad, Mar Caribe colombiano. Bulletin of Marine and Coastal Research.

[B12494946] Moseley Henry Nottidge (1877). On *Stylochuspelagicus*, a new species of pelagic planarian, with notes on other pelagic species, on the larval forms of *Thysanzoon*, and of a gymnosomatous pteropod. Journal of Cell Science.

[B12493542] Muñoz Bautista A. N., Aké Castillo J. A., Granados Barba A., Pérez España H., Granados Barba A., Ortiz Lozano L., Salas Monreal D., González Gándara C. (2015). Aportes al conocimiento del Sistema Arrecifal Veracruzano: hacia el Corredor Arrecifal del Suroeste del Golfo de México.

[B12700621] Oya Yuki, Kajihara Hiroshi (2020). Molecular phylogenetic analysis of Acotylea (Platyhelminthes: Polycladida). Zoological Science.

[B12492755] Plehn Marianne, Hensen Victor (1896). Ergebnisse der in dem Atlantischen Ocean von mitte Juli bis anfang November 1889 ausgeführten Plankton-Expedition der Humboldt-Stiftung. Auf Frund von gemeinschaftlichen Untersuchungen einer Reihe von Fach-Forschern.

[B12495047] Prudhoe Stephen (1944). On some polyclad turbellarians from the Cayman Islands. Annals and Magazine of Natural History.

[B12493490] Prudhoe Stephen (1985). A monograph on polyclad turbellaria.

[B12495029] Quiroga Sigmer Y., Bolaños D. Marcela, Litvaitis Marian K. (2004). A checklist of polyclad flatworms (Platyhelminthes: Polycladida) from the Caribbean coast of Colombia, South America. Zootaxa.

[B12492768] Smetacek Victor, Zingone Adriana (2013). Green and golden seaweed tides on the rise. Nature.

[B12493584] Sonnenberg Rainer, Nolte Arne W., Tautz Diethard (2007). An evaluation of LSU rDNA D1-D2 sequences for their use in species identification. Frontiers in Zoology.

[B12495411] Trott Tammy M., McKenna Sheila, Pitt Joanna, Hemphill Arlo, Ming Frederick, Rouja Philippe, Gjerde Kristina, Causey Billy, Earle Sylvia (2011). Efforts to enhance protection of the Sargasso Sea.

[B12492741] van Tussenbroek Brigitta I., Monroy-Velázquez L. Verónica, García-Sánchez Marta, Ruiz-Fernández Ana Carolina, Valencia-Castañeda Gladys, Paéz-Osuna Federico, Arenas Pablo, Rojas-González R. Isaac, Gracia Adolfo (2024). Biochemistry and associated fauna of holopelagic *Sargassum* spp. in the Caribbean Sea. Marine Biology.

[B12492724] van Tussenbroek Brigitta I., Monroy-Velázquez L. Verónica, Rodríguez Dení, Suescún-Bolívar L. Parmenio, Thomé Patricia E., Cerqueda-García Daniel, García-Maldonado José Q., Martínez-López Isis G., López-Portillo José Antonio, Barba-Santos M. Guadalupe, Gómez-Reali Miguel Angel, Escalante-Mancera J. Edgar (2024). Monitoring drift and associated biodiversity of nearshore rafts of holopelagic *Sargassum* spp. in the Mexican Caribbean. Aquatic Botany.

[B12492778] Vázquez-Delfín Erika, Robledo Daniel, Freile-Pelegrín Yolanda (2024). Temporal characterization of *Sargassum* (Sargassaceae, Phaeophyceae) strandings in a sandy beach of Quintana Roo, Mexico: Ecological implications for coastal ecosystems and management. Thalassas: An International Journal of Marine Sciences.

[B12492713] Wang Mengqiu, Hu Chuanmin, Barnes Brian B., Mitchum Gary, Lapointe Brian, Montoya Joseph P. (2019). The great Atlantic *Sargassum* belt. Science.

[B12496538] Williams Gary C., Van Syoc Robert (2007). Methods of preservation and anesthetization of marine invertebrates. The Light and Smith Manual.

